# Supplementary material for: Dietary Carbohydrates and Insulin Resistance in Adolescents from Marginalized Areas of Chiapas, México
Source: Nutrients. 2019 Dec 16;11(12):3066. doi: 10.3390/nu11123066 (PMC6950049; doi:10.3390/nu11123066)
Supplement: Supplementary file 1 [file nutrients-11-03066-s001.pdf]

Table S1. Measures of quality and Hosmer–Lemeshow tests for logistic regression models of associations between categories of carbohydrate intake variables and insulin resistance (HOMA-IR >3.16) in adolescents from Chiapas, Mexico.

Factor variables		Insulin resistance (HOMA-IR > 3.16)			
		OR (95% CI)	OR (95% CI)	OR (95% CI)	OR (95% CI)
Total Carbohydrates[ab]		Model 1	Model 2[c]	Model 3[d]	Model 4[e]
**Males**					
Sensitivity		47.37%	47.37%	36.84%	42.11%
Specificity		68.48%	77.17%	81.52%	79.35%
Hosmer–Lemeshow test	p-value	NA*	0.296	0.296	0.153
Females					
Sensitivity		62.96%	88.89%	85.19%	81.48%
Specificity		65.82%	56.96%	56.96%	68.35%
Hosmer–Lemeshow test	p-value	NA*	0.051	0.090	0.376
Dietary Fiber[a]		Model 1	Model 2[f]	Model 3[g]	Model 4[h]
Sensitivity		54.35%	71.74%	71.74%	67.39%
Specificity		71.93%	63.16%	64.91%	66.67%
Hosmer–Lemeshow test	p-value	NA*	0.402	0.502	0.112
Total Sugars[a]		Model 1	Model 2[f]	Model 3[g]	Model 4[i]
Sensitivity		78.26%	67.39%	60.87%	69.57%
Specificity		36.26%	61.40%	63.16%	63.74%
Hosmer–Lemeshow test	p-value	NA*	0.295	0.262	0.168
Dietary GI		Model 1	Model 2[f]	Model 3[j]	Model 4[k]
Sensitivity		43.48%	60.87%	71.74%	67.39%
Specificity		69.59%	56.14%	53.80%	63.16%
Hosmer–Lemeshow test	p-value	NA*	0.274	0.294	0.113
Dietary GL[a]		Model 1	Model 2[f]	Model 3[j]	Model 4[l]
Sensitivity		41.30%	63.04%	71.74%	69.57%
Specificity		68.42%	56.73%	54.39%	60.82%
Hosmer–Lemeshow test	p-value	NA*	0.238	0.183	0.102

[a] Energy-adjusted using the residuals method. [b] Interaction term $p < 0.05$. [c] Adjusted for age (years). [d] Adjusted for age (years) and BF% (normal/high). [e] Adjusted for age (years), BF% (normal/high), energy-adjusted dietary fiber intake (g/day) and energy-adjusted MUFAs intake (g/day). [f] Adjusted for sex (males/females) and age (years). [g] Adjusted for sex (males/females), age (years) and BF% (normal/high). [h] Adjusted for sex (males/females), age (years), BF% (normal/high), energy-adjusted MUFAs intake (g/day) and total energy intake (kcal/day). [i] Adjusted for sex (males/females), age (years), BF% (normal/high) and energy-adjusted MUFAs intake (g/day). [j] Adjusted for sex (males/females), age (years), geographic area (rural/urban) and BF% (normal/high). [k] Adjusted for sex (males/females), age (years), geographic area (rural/urban), BF% (normal/high), energy-adjusted MUFAs intake (g/day) and total energy intake (kcal/day). [l] Adjusted for sex (males/females), age (years), geographic area (rural/urban), BF% (normal/high) and energy-adjusted MUFAs intake (g/day). HOMA-IR, homeostasis model assessment of insulin resistance; T, tertiles; BF%, body fat percentage; MUFAs, monounsaturated fatty acids; GI, glycemic index; GL, glycemic load.

Table S2. Measures of quality and Hosmer–Lemeshow tests for logistic regression models of associations between insulin resistance (HOMA-IR >2.97) and categories of carbohydrate intake variables in adolescents from Chiapas, Mexico.

**Factor variables**		**Insulin resistance (HOMA-IR > 2.97)**			
		**OR (95% CI)**	**OR (95% CI)**	**OR (95% CI)**	**OR (95% CI)**
Total Carbohydrates[ab]		Model 1	Model 2[b]	Model 3[c]	Model 4[d]
Sensitivity		74.00%	60.00%	64.00%	68.00%
Specificity		35.33%	55.09%	58.08%	62.28%
Hosmer–Lemeshow test	p-value	NA*	0.215	0.223	0.109
Dietary Fiber[a]		Model 1	Model 2[b]	Model 3[c]	Model 4[e]
Sensitivity		84.00%	70.00%	74.00%	72.00%
Specificity		38.32%	59.28%	58.08%	60.48%
Hosmer–Lemeshow test	p-value	NA*	0.121	0.168	0.197
Total Sugars[a]		Model 1	Model 2[b]	Model 3[c]	Model 4[f]
Sensitivity		80.00%	62.00%	66.00%	74.00%
Specificity		37.13%	61.08%	61.08%	60.48%
Hosmer–Lemeshow test	p-value	NA*	0.350	0.320	0.329
Dietary GI		Model 1	Model 2[b]	Model 3[c]	Model 4[g]
Sensitivity		78.00%	60.00%	64.00%	66.00%
Specificity		37.13%	55.69%	59.28%	60.48%
Hosmer–Lemeshow test	p-value	NA*	0.291	0.336	0.200
Dietary GL[a]		Model 1	Model 2[b]	Model 3[h]	Model 4[i]
Sensitivity		72.00%	64.00%	60.00%	68.00%
Specificity		34.73%	59.88%	62.87%	65.87%
Hosmer–Lemeshow test	p-value	NA*	0.269	0.306	0.166

[a] Energy-adjusted using the residuals method. [b] Adjusted for sex (males/females) and age (years). [c] Adjusted for sex (males/females), age (years) and BF% (normal/high). [d] Adjusted for sex (males/females), age (years), BF% (normal/high) and energy-adjusted dietary fiber intake (g/day). [e] Adjusted for sex (males/females), age (years), BF% (normal/high) and energy-adjusted SFAs intake (g/day). [f] Adjusted for sex (males/females), age (years), BF% (normal/high) and lipids (% energy). [g] Adjusted for sex (males/females), age (years), BF% (normal/high) and energy-adjusted MUFAs intake (g/day). [h] Adjusted for sex (males/females), age (years), waist circumference (normal/high). [h] Adjusted for sex (males/females), age (years), waist circumference (normal/high) and energy-adjusted dietary fiber intake (g/day). BF%, body fat percentage; SFAs, saturated fatty acids.

Table S3. Measures of quality and Hosmer–Lemeshow tests for logistic regression models of associations between categories of carbohydrate intake variables and elevated fasting insulin concentration (≥14.38 μU/mL) in adolescents from Chiapas, Mexico.

Factor variables		Elevated fasting insulin concentration (≥14.38 μU/mL)			
		OR (95% CI)	OR (95% CI)	OR (95% CI)	OR (95% CI)
Total Carbohydrates[ab]		Model 1	Model 2[b]	Model 3[c]	Model 4[d]
Sensitivity		78.72%	65.96%	65.96%	70.21%
Specificity		36.47%	55.88%	63.53%	62.35%
Hosmer–Lemeshow test	p-value	NA*	0.117	0.194	0.179
Dietary Fiber[a]		Model 1	Model 2[b]	Model 3[c]	Model 4[e]
Sensitivity		55.32%	70.21%	68.09%	72.34%
Specificity		72.35%	63.53%	66.47%	65.88%
Hosmer–Lemeshow test	p-value	NA*	0.031	0.070	0.076
Total Sugars[a]		Model 1	Model 2[b]	Model 3[c]	Model 4[d]
Sensitivity		80.85%	68.09%	70.21%	70.21%
Specificity		37.06%	63.53%	62.94%	65.29%
Hosmer–Lemeshow test	p-value	NA*	0.249	0.218	0.374
Dietary GI		Model 1	Model 2[b]	Model 3[f]	Model 4[g]
Sensitivity		44.68%	65.96%	65.96%	70.21%
Specificity		70.00%	58.24%	64.12%	65.88%
Hosmer–Lemeshow test	p-value	NA*	0.158	0.311	0.190
Dietary GL[a]		Model 1	Model 2[b]	Model 3[f]	Model 4[h]
Sensitivity		40.43%	65.96%	68.09%	72.34%
Specificity		68.24%	58.24%	65.88%	65.29%
Hosmer–Lemeshow test	p-value	NA*	0.159	0.397	0.168

[a] Energy-adjusted using the residuals method. [b] Adjusted for sex (males/females) and age (years). [c] Adjusted for sex (males/females), age (years) and BMI (normal/overweight or obesity). [d] Adjusted for sex (males/females), age (years), BMI (normal/overweight or obesity) and energy-adjusted MUFAs intake (g/day). [e] Adjusted for sex (males/females), age (years), BMI (normal/overweight or obesity), energy-adjusted MUFAs intake (g/day) and total energy intake (kcal/day). [f] Adjusted for sex (males/females), age (years), Mother's language (Spanish/Indigenous) and BMI (normal/overweight or obesity). [g] Adjusted for sex (males/females), age (years), mother's language (Spanish/Indigenous), BMI (normal/overweight or obesity) and energy-adjusted dietary fiber intake (g/day). [h] Adjusted for sex (males/females), age (years), Mother's language (Spanish/Indigenous), energy-adjusted dietary fiber intake (g/day) and total energy intake (kcal/day). BMI, body mass index.
